# Fluazuron orally administered to guinea pigs: pharmacokinetic and efficacy against *Amblyomma sculptum*

**DOI:** 10.1186/s13071-022-05325-4

**Published:** 2022-06-10

**Authors:** Debora Azevedo Borges, Yara Peluso Cid, Viviane de Sousa Magalhães, Melina Cardilo Campos Alves, Thais Paes Ferreira, Isabelle Vilela Bonfim, Emily Andressa Santos Lima, Juliana Pereira de Freitas, Fabio Barbour Scott

**Affiliations:** 1 Animal Parasitology Department, Veterinary Institute, Federal Rural University of Rio de Janeiro, Seropédica, RJ Brazil; 2 Pharmaceutical Science Department, Health and Biological Science Institute, Federal Rural University of Rio de Janeiro, Seropédica, RJ Brazil

**Keywords:** Insect growth regulators, Tick control, *Cavia porcellus*, Ectoparasites, Capybaras, Bioavailability

## Abstract

**Background:**

Brazilian spotted fever (BSF), the most lethal tick-borne disease in the Western Hemisphere, is caused by the bacterium *Rickettsia rickettsii* and transmitted by the bite of *Amblyomma sculptum*. Capybaras are considered primary hosts of this tick and amplifier hosts of *R. rickettsii*, generating new infected lineages of *A. sculptum* in BSF-endemic areas. To define a possible treatment regimen for controlling the tick *A. sculptum* in capybaras, the aim of this study was to establish an effective fluazuron (FLU) dose to control *A. sculptum* larvae in artificially infested guinea pigs.

**Methods:**

In Study I (pharmacokinetic and pharmacodynamic analysis), 24 guinea pigs were divided into four equal groups: control group (CG; untreated) and treated groups receiving FLU administered by gavage in three doses: G1—1 mg/kg, G2—5 mg/kg and G3—10 mg/kg, once a day for 15 days (d0 to d + 14). Blood samples were collected from the animals of the treated groups before and at d + 1, + 2, + 4, + 7, + 15 and + 21. The guinea pigs were artificially infested at d + 7 with *A. sculptum* larvae, and specimens were recovered at d + 11 to d + 14 and kept in a climatized chamber for 14 days. In Study II (evaluation of pharmacokinetic parameters), one group of eight animals received FLU administered by gavage in a single dose of 10 mg/kg, and blood samples were collected before and on day 0 (8 h after treatment), + 1, + 4, + 7, + 15, + 21 and + 28 after single FLU administration. FLU was analyzed in plasma samples by high-performance liquid chromatography with ultraviolet detection.

**Results:**

FLU plasma concentrations increased quickly, indicating rapid absorption, and decreased slowly. Some larvae from all treated groups exhibited morphological and behavioral changes. FLU interfered in molting, and the efficacy obtained was 100% for all treated groups.

**Conclusions:**

The results offer promising perspectives for the development of a palatable feed cube containing FLU for free-living capybaras to control *A. sculptum* and also to prevent BSF in areas where capybaras have been shown to play a primary role.

## Background

*Amblyomma sculptum* belongs to a complex of five other tick species, of which it has the widest distribution in Brazil [1]. This arthropod species has low parasitic specificity, mainly in immature stages, being able to infest several vertebrate host species, including humans as accidental hosts. However, capybaras, tapirs and horses are considered its preferred hosts [2].

A factor that contributes to the excessive infestation of these ticks in the environment is the population imbalance of capybaras in certain areas, haing an ecological and public health impact [3]. Capybaras are the largest living rodent [4] and have the capacity to adapt to anthropic habitats, with important reproductive potential. They also live in large groups and do not have predators. These factors have led to a considerable increase in the population density of capybaras, and consequently of ticks [5].

Brazilian Spotted Fever (BSF) is an infectious disease with high case fatality risk, caused by the bacterium *Rickettsia rickettsii* [6] and transmitted through the bite of ticks, mainly *A. sculptum* [7].

To obtain effectiveness in controlling BSF, the control of ticks such as *A. sculptum* is necessary to reduce their number in both animals and the environment. The immature stages of ticks (especially larvae) are more sensitive to chemical control measures than adults [8]. Therefore, reducing the presence of ticks in the immature phase is more effective than controlling the number of adult ticks. Furthermore, the correct use of acaricides in livestock is necessary to control environments infested by *A. sculptum*, since the concomitant presence of wild animals such as small mammals is common. The major issue in that case is that these wild animals can act as hosts of ticks in the immature stages, becoming keepers and dispersers of these arthropods in the environment [9].

Despite the severity of BSF, some in vitro studies have been published on the efficacy of *A. sculptum* control [10–12], and they have not been reported in in vivo studies.

The main chemical groups for tick control applied on hosts are carbamates, organophosphates, amidines, pyrethroids, macrocyclic lactones and phenylpyrazoles [13]. Fluazuron (FLU) belongs to a class of insect growth regulators (IGRs), and it is a chitin synthesis inhibitor [14]. FLU acts by interfering with molting and hatching [15] and is widely used to control *Rhipicephalus microplus* on cattle [16–18].

Few studies have described the oral administration of FLU to control ectoparasite in animals. For example, it was considered viable to control *Sarcoptes scabei* in pigs [19]. In addition, the administration of FLU in feed cubes proved to be viable for the control of fleas on wild rodents [20, 21].

To enable future development of feed cubes containing fluazuron for the control of the tick *A. sculptum* in capybaras, the aim of this study was to establish the bioavailability and the effective dose of FLU administered orally for the control of *A. sculptum* larvae using artificially infested guinea pigs (*Cavia porcellus*).

## Methods

### Animals

Thirty-two clinically healthy guinea pigs (16 males and 16 females), 6–8 months old, weighing 0.6–1 kg, were included in the study. The animals had not been treated with ectoparasiticides in the 3 months before treatment. All animals were housed individually in cages whose dimensions were 0.60 m (height) × 1.2 m (width) × 0.60 m (depth), placed on a masonry floor, with supply of freshwater ad libitum and dry feed for guinea pigs twice a day, according to the weight and need of each individual. The temperature of the room where the animals were kept was controlled by air conditioning, keeping it at 21 ± 1 °C. Also, environmental enrichment measures were adopted to reduce stress caused by the confinement and management necessary to conduct the study.

### Groups and treatment

Two studies were carried out, as follows:

Study I: This study aimed to determine the influence of FLU doses on plasma concentrations and efficacy. For pharmacokinetic and pharmacodynamic (PK/PD) analysis, 24 animals were divided into four groups (six animals/group): control group (CG; untreated) and groups 1 (G1), 2 (G2) and 3 (G3), receiving FLU administered by gavage in three doses, G1—1 mg/kg body weight, G2—5 mg/kg body weight and G3—10 mg/kg body weight, once a day for 15 days (d0 to d + 14). The guinea pigs were randomized by sex and weight. A decreasing list for each sex was prepared with the weight of each animal. Then the animals were divided into four groups (CG, G1, G2 and G3), and a lottery was conducted from the heaviest to the lightest animal, allocating one in each group, and so on, until attaining six replicates in the four groups, with three males and three females in each group.

Study II: This study aimed to perform descriptive pharmacokinetics. Determination of the pharmacokinetic parameters of FLU was obtained by evaluating the single-dose, compartmental model study. For evaluation of pharmacokinetic parameters, one group with eight animals received FLU administered by gavage at a single dose of 10 mg/kg.

### Pharmacokinetic analysis

Study I: Blood was collected from the animals of G1, G2 and G3 in heparin tubes by jugular venipuncture before and after the first treatment with FLU on days + 1, + 2, + 4, + 7, + 15 and + 21.

Study II: Blood samples were collected before and on days 0 (8 h after treatment), + 1, + 4, + 7, + 15, + 21 and + 28 after administration of a single FLU dose.

In both studies the plasma was obtained by centrifugation at 756 *g* for 10 min at 4 °C and was stored at − 20 °C until analysis.

Pharmacokinetic parameters were determined using the PK solver program (Microsoft Excel®, Redmond, WA, USA), using the non-compartmental model of extravascular administration. All PK parameters were calculated using the individual plasma concentration versus time data. The maximum measured concentration for a particular animal (Cmax) and the time from dosing to the maximum concentration (Tmax) were measured individually. The area under the curve from zero to last time t (AUC0-t) was calculated using the linear trapezoidal method and extrapolated to infinity (AUC0-∞).

Results are expressed as arithmetic mean ± standard deviation (SD). Data were statistically analyzed by one-way ANOVA followed by the Tukey test for multiple comparisons using GraphPad Prism 6.0 with 95% significance (*p* ≤ 0.05).

### Analytical procedures

The plasma concentrations of FLU were analyzed by high-performance liquid chromatography (HPLC) with ultraviolet detection and solid-phase extraction (SPE) according to the procedure described by Ferreira et al. [22], adapted and optimized for guinea pig plasma. Plasma samples were subjected to SPE clean-up using Discovery 18-LT extraction cartridges (500 mg, 3 ml) (Supelco, USA) connected to a Visiprep SPE vacuum manifold (Supelco, USA), using acetonitrile as eluent solvent. The eluate was evaporated to dryness and reconstituted in 100 μl acetonitrile. The chromatographic separation was performed using a C18 column (Kromasil, 3.5 µm; 4.6 × 100 mm; Tedia, Rio de Janeiro, Brazil), preceded by a C18 guard column (Kromasil, 3.5 µm; 4.6 × 10 mm; Tedia, Rio de Janeiro, Brazil), both maintained at 25 °C. The mobile phase consisted of acetonitrile: water (80:20, v/v) with a flow rate of 1.0 ml/min. The UV wavelength was set at 260 nm, and the injection volume was 20 µl.

### Efficacy studies

The larvae of *A. sculptum* used in the experiment were obtained from colonies maintained in rabbits in the Laboratory for Experimental Chemotherapy in Veterinary Parasitology of Federal Rural University of Rio de Janeiro.

To evaluate whether FLU could interfere with the ecdysis of engorged *A. sculptum* larvae, the guinea pigs of the treated groups received FLU by gavage at three different doses once a day on experimental days 0 to 14 (Study I).

On d + 6, a calico bag was attached on the back [23] of each animal (including the treated group) with Unna’s paste [24] and adhesive plaster. All guinea pigs (tick-bite naïve) were infested with approximately 1000 unfed *A. sculptum* larvae on d + 7 (the number of larvae used for infestation process was not determined by individual counting but taken from egg mass weights, with a count of eggs by gram, whereas the egg hatching percentage was pre-determined).

The first observation was performed after 24 h, the period needed for larval attachment. Daily, from d + 8 to d + 14, the calico bags were inspected and naturally detached engorged ticks were collected, counted and immediately transferred to an incubator at 27 ºC and 85% RH, where they were kept. After this, all ticks were evaluated and counted as alive or dead.

Statistical analysis of the number of engorged larvae detached was performed regarding data normality, using the Shapiro-Wilk test, between the experimental groups.

The average percentage data of molting from engorged larvae to nymph were used to determine whether there was a significant difference using ANOVA (a criterion) between the experimental groups. In all analyses a significance percentage ≥ 95% was considered. Analyses were performed using the statistical program BioEstat 5.3 [25, 26].

## Results

### Pharmacokinetic analysis

Clinically, no adverse reactions were observed in any of the guinea pigs treated with FLU administered by gavage. In all experimental groups, plasma concentrations of FLU were quantified at all post-treatment sampling times.

The mean values of FLU plasma concentration of each group (Study I) are shown in Table 1.

**Table 1:** Mean ± SD of plasma concentration of fluazuron (ng.ml^−1^) in guinea pigs treated orally in multiple doses with doses of 1 mg/kg, 5 mg/kg and 10 mg/kg during the treatment period

Groups	Evaluation days					
	Day + 1	Day + 2	Day + 4	Day + 7	Day + 15	Day + 21
G1 (1 mg/kg)	50.87 ± 3.55^a^	298,99 ± 141.45^a,b^	400.67 ± 160.53^a,b^	98.12 ± 19.89^a,c^	157.83 ± 102.64^a,b,c^	52.29 ± 29.43^a,c^
G2 (5 mg/kg)	164.44 ± 9.78^b^	226.96 ± 110.04^b^	509.99 ± 366.23^b^	250.20 ± 79.95^b^	348.37 ± 158.47^b^	161.40 ± 66.62^b^
G3 (10 mg/kg)	377.77 ± 46.62^b^	525.00 ± 38.50^b^	701.43 ± 423.42^b^	519.02 ± 273.33^b^	499.73 ± 220.76^b^	293.84 ± 151.17^b^

^a^values of G1 differ from G2 and G3 in all evaluation days (significance level of 5%)

^b^values of G2 and G3 do not differ from each other in all evaluation days; on the average line of G1 +2, +4 and +15 do not differ from each other (significance level of 5%)

^c^on the average line of G1 +7, +15 and +21 do not differ from each other (significance level of 5%)

Daily dose administrations for 15 days generated concentrations of FLU in plasma ranging from 50.87 to 400.67 ng.ml^−1^ at the lowest dose (1 mg/kg), from 164.44 to 509.99 ng.ml^−1^ at the medium dose (5 mg/kg) and from 293.84 to 701.43 ng.ml^−1^ at the highest dose (10 mg/kg). The lowest dose presented lowest concentration values in plasma compared with medium and highest dose, since the statistical analyses showed significant difference (*p* < 0,005) at the first day of evaluation (Day + 1). However, for medium and highest doses, although increasing the dose resulted in an increase of FLU plasma concentration, multiple doses (once a day for 15 days) did not lead to an increase of FLU concentrations over the course of treatment at both doses—no significant differences were observed between the evaluation days.

**Fig. 1:**
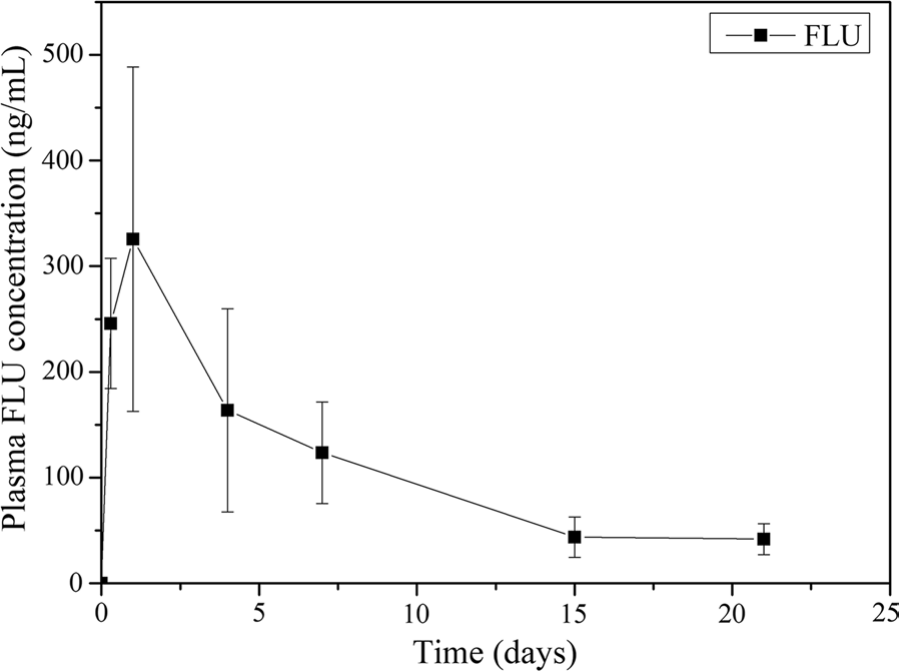
Mean ± SD plasma concentration of fluazuron following orally administration of fluazuron (10 mg/kg) to guinea pigs (*n* = 8)

In Study II, the FLU plasma concentration versus time curves after single treatment are shown in Fig. 1. FLU plasma concentrations increased quickly, indicating rapid absorption, reaching C_max_ of 356.55 ± 133.75 ng/ml at 0.77 ± 0.35 days (T_max_), with absorption intervals (AUC0-t) of 2327.99 ± 673.39, ng/ml*d, and slow elimination with t_1/2_ of 6.94 ± 1.47 (Table 2).

**Table 2:** Pharmacokinetic parameters of fluazuron following orally administration of fluazuron (10 mg/kg) to guinea pigs (*n* = 8)

Pharmacokinetic parameters	Arithmetic mean ± SD
C_max_ (ng/ml)	356.55 ± 133.75
t_max_ (d)	0.77 ± 0.35
AUC_0-t_ (ng.d/ml)	2327.99 ± 673.39
AUC_0-∞_ (ng.d/ml)	2768.13 ± 708.61
t_½_ (d)	6.94 ± 1.47

*tmax* time to reach peak plasma concentration, *Cmax* peak plasma concentration, *AUC* area under the (zero moment) curve from time 0 to the last detectable concentration, *t1/2* terminal half-life, *AUC* area under the (zero moment) curve from time 0 to infinity

### Efficacy studies

All data regarding efficacy can be seen in Table 3. The ticks detached from d + 11 to d + 14, and the means of recovery values of engorged specimens were 266.8 (± 54.6) for CG, 419.2 (± 276.0) for G1, 427.2 (± 244.6) for G2 and 371.3 (± 203.1) for G3 (Table 3).

**Table 3:** Evaluation of detached and molting process of engorged larvae of *Amblyomma sculptum* recovered from artificially infested guinea pigs for control and fluazuron treated groups (at 1, 5 and 10 mg/kg)

Group	N of engorged larvae detached	Larvicidal efficacy (%)	N of nymphs that have molted	Nymphs that have molted (%)	Molting process inhibition (%)
CG	266.8^1^ ± 54.6^a^*	–	247.0^1^ ± 55.1	92.4^1^ ± 7.0^a^	–
	(189–359)^2^		(176–330)^2^	(77.5–99.2)^2^	
G1	419.2^1^ ± 276.0^a^**	0	0^1^	0^1, b^	100
	(132–777)^2^		(0)^2^	(0)^2^	
G2	427.2^1^ ± 244.6^a^**	0	0^1^	0^1, b^	100
	(227–788)^2^		(0) ^2^	(0)^2^	
G3	371.3^1^ ± 203.1^a^**	0	0^1^	0^1, b^	100
	(101–598)^2^		(0) ^2^	(0)^1, 2^	

^a^In column of engorged lavae detached the groups do not differ from each other (p=0.8123) and in the column of nymphs that have molted (%) the control group differ from the treated groups (p<0.0001)

^b^n column of nymphs that have molted (%) the treated groups do not differ from each other

*CG* Control group, *G1* 1 mg/kg, *G2* 5 mg/kg, *G3* 10 mg/kg

^*^Normal morphology and behavior

^**^Engorged larvae shriveled and darker

^1^Arithmetic mean

^2^Minimum and maximum values

The results showed that the data had nonparametric distribution. Data were log_10_ transformed for normalization. The Shapiro-Wilk test was used, noting that the data reached a normal distribution. Later, ANOVA (a criterion) was used to compare the mean number of engorged larvae recovered between the experimental groups. The results showed no significant difference (*p* = 0.8123) for engorged larvae detached.

Immediately after detachment, some larvae from all treated groups, mainly G3, although alive, exhibited morphological and behavioral changes, such as stunted size, elliptical shape, fragile integument and lethargy.

The mean molting percentage for the control group was 92.4% (± 7), while for all treated groups it was 0 (100% efficacy). There was a significant difference between the mean molting percentage of the control group and the three medicated groups (*p* < 0.0001).

After the molting period, all larvae from the treated groups were shriveled and darker. In the control group, no morphological or behavioral alterations were observed.

## Discussion

In this study, we chose the guinea pig (*C. porcellus*), a close relative of the capybaras (*Hydrochaeris hydrochaeris*), as the experimental model. Both belong to the Caviidae family and have similar behavior and physiology [27]. These preliminary tests were conducted in guinea pigs because of the facility of handling, since they are smaller and more docile than capybaras.

Fluazuron has been used in the control of the tick *Rhipicephalus microplus* [16–18], but few controlled studies are available on the control of heteroxenous ticks, and in all cases the drug was applied topically [28–30].

No statistical difference in the count of detached engorged larvae between the groups was observed. However, despite this absence of the knockdown effect of FLU used as an acaricide, 14 days after engorged larvae incubation, all larvae from treated groups were dead. Furthermore, the engorged larvae, mainly from G3, exhibited morphological and behavioral changes. Oliveira et al. [28], in a study of rabbits treated with different doses of FLU (1.25 to 150 mg/kg, poured on) to control *R. sanguineus*, observed the same morphological and behavioral changes in engorged nymphs in groups that received doses of 10 mg/kg. Probably the continued use and the route (oral instead of topical) of FLU applied to the animals of this study influenced the alterations observed even in the group receiving the lowest dose (1 mg/kg).

To determine whether there was a residual effect of FLU, other infestations of the same animals would be necessary, but since there are several studies proving that guinea pigs acquire resistance to ticks after a first infestation [31–35], we chose not to infest the animals again.

Data obtained from Study I showed an increase in plasma concentration of FLU with increasing dose on day + 1. However, from day + 4 until the last day of evaluation (+ 21), mean plasma concentrations did not differ significantly between groups. Efficacy results showed that even the lowest dose achieved 100% efficacy against larval molting. These results can allow choice of a dose between 1 and 10 mg/kg (orally) in further studies to control *A. sculptum* on capybaras.

In Study I, on d + 7, G1 had plasma FLU concentration of 98.12 ng.ml^−1^ (± 19.89), similar to the result of Study II on the same experimental day (d + 7). Since in the multiple dose study (Study I), 100% efficacy was obtained against conclusion of molting at plasma concentrations of FLU close to 100 ng.ml^−1^, perhaps treatment every 7 days with oral FLU dose of 10 mg/kg would be sufficient to control *A. sculptum* in guinea pigs and possibly in capybaras as well.

Pasay et al. [19] administered FLU orally for 7 days (10 mg/kg/day) to control *S. scabei* infestation in three pigs and achieved plasma peaks of 300–800 ng.ml^−1^ on day 7, similar to the result observed in Study I, with average of 701.43 ng.ml^−1^ on day 4 (plasma peak) in animals treated with 10 mg/kg/day. Although those authors did not calculate the pharmacokinetic parameters, we observed the same behavior, with rapid absorption and slow elimination until no detection on day 28.

The use of FLU as ectoparasiticide and its pharmacokinetic parameters were described by Lopes et al. [18] and Ferreira et al. [22] for pour-on formulations in cattle (2.5 mg/kg, single dose). However, according to Flajs and Grabnar [36], although the comparison of pharmacokinetic parameters between different species and with different administration routes is not recommended, it is still possible to compare the average time when levels of the drug can be detected in the blood plasma.

In Study II, we observed the same pattern of quick absorption and slow elimination of FLU as reported by Lopes et al. [18] and Ferreira et al. [22].

No data exist on effective systemic FLU dosages for the control of *A. sculptum* in guinea pigs or capybaras, and there are only a few studies in which FLU was given orally to animals for control of fleas [20, 21], mites [19] and ticks [20]. The latter research group conducted a field trial of FLU in woodrats against fleas and ticks, but it did not reduce tick counts during a year of evaluation, with doses from 1 to 40 mg/kg given in feed cubes. However, the authors attributed the absence of count reduction to the extended life cycle of the species of ticks in the studied area (e.g. *Ixodes pacificus* can take as long as 3 years to complete its life cycle). It is possible that in a controlled study, the authors would have observed the results they expected.

The population imbalance of capybaras in certain areas is indicated as the main cause of excessive tick infestation, causing an important ecological impact, with risk to public health due to the transmission of the bacterium *R. rickettsii* by the tick *A. sculptum*. All attempts to control tick infestations in environments where there are capybaras have been based on either removing these animals or placing fences around areas frequented by capybaras in an attempt to isolate them from people who visit parks [37] or placing horses treated with ectoparasiticides in those areas (acting as traps) [38].

However, the results obtained have shown that the control of spotted fever is highly complex because of the many factors involved, requiring strategic control [39]. Therefore, actions are needed to enable population control of capybaras, environmental control of the evolutionary forms of *A. sculptum*, isolation of the most critical areas of parks to minimize human contact with ticks and, last but not least, the search for tools that enable tick control at the time they are parasitizing capybaras.

The topical application of products with acaricidal effect on capybaras is unfeasible because of the need to capture these animals and the fact they remain in the water for many hours. Based on the satisfactory results obtained in this study, combined with methods already employed to control ticks in free-living rodents, our results allow future tests with the use of fluazuron in paraffin blocks (feed cubes) to control the tick *A. sculptum* in capybaras.

## Conclusions

The results of this study indicate that the plasma availability of FLU administered orally in guinea pigs is effective against engorged *A. sculptum* larvae, bringing perspectives for the development of palatable feed cubes containing FLU for control of *A. sculptum* on free-living capybaras and also to prevent BSF in areas where capybaras have been shown to play a primary role.

## Data Availability

Supporting data for the conclusions of this article are included within the article and its additional files. The raw datasets used and analyzed during this study are available upon reasonable request.
